# Genome Sequence of Human Papillomavirus 23 Strain HPV-23/Lancaster/2015

**DOI:** 10.1128/genomeA.00257-17

**Published:** 2017-05-18

**Authors:** Kate V. Atkinson, Lisa A. Bishop, Glenn Rhodes, Nicolas Salez, Neil R. McEwan, Matthew J. Hegarty, Julie Robey, Nicola Harding, Simon Wetherell, Robert M. Lauder, Roger W. Pickup, Mark Wilkinson, Derek Gatherer

**Affiliations:** a Division of Biomedical & Life Sciences, Faculty of Health & Medicine, Lancaster University, Lancaster, United Kingdom; b Royal Lancaster Infirmary, Lancaster, United Kingdom; c Centre for Ecology & Hydrology, Lake Ecosystems Group, Lancaster Environment Centre, Lancaster University, Lancaster, United Kingdom; d UMR_D 190, Emergence des Pathologies Virales, Aix-Marseille University, Marseille, France; e Institute of Biological, Environmental & Rural Sciences, Aberystwyth University, Aberystwyth, United Kingdom; f Queen Square Medical Practice, Lancaster, United Kingdom

## Abstract

The genome of human papillomavirus type 23 (HPV-23; family *Papillomaviridae*, genus *Betapapillomavirus*, species *Betapapillomavirus 2*, type 23) was assembled by deep sequencing from nasopharyngeal swabs. The assembled genome is 2.7% divergent over its full length from the single complete genome of HPV-23 in GenBank (accession no. U31781). We named the strain HPV-23/Lancaster/2015.

## GENOME ANNOUNCEMENT

The family *Papillomaviridae* consists of more than 120 viral types divided into 49 genera (1). Within the genus* Betapapillomavirus*, type 23 of species *Betapapillomavirus 2* (HPV-23) is represented in GenBank by a single complete genome (2) composed of a circular double-stranded DNA of 7.3 kb (accession no. U31781), as well as two shorter fragments (3, 4). HPV-23 is a clinically significant papillomavirus, having been implicated in epidermodysplasia verruciformis (2), skin cancer (5), ocular syringoma (6), nongenital seborrhoeic keratosis (7), breast cancer (8), and toenail onycholysis (9).

Volunteers were recruited from a general practice surgery and a general hospital in Lancaster, UK (54.05°N, 2.80°W). Nasopharyngeal swabs were taken between 16 December 2014 and 25 February 2015. Ethical approval was granted by the UK National Research Ethics Service, reference 14/LO/1634, NIHR Clinical Research Network (UKCRN) portfolio, ID 17799. All methods were carried out in accordance with the relevant guidelines and regulations.

Pooled RNA from 51 swabs was deep sequenced using an Illumina Nextera XT library and HiSeq 2500 system (SRA GenBank accession no. SRP092324). An HPV-23 genome was assembled using Bowtie 1.1.1 (10) and BWA 0.7.12-r1039 (11), with U31781 as the template. The assembled genome is 7,317 bases in length, differing from U31781 by 188 substitutions (2.7%). A 6-base deletion in the new genome starts at position 5418, within the L2 gene. A further single-base deletion occurs at position 7193, in the 3′ direction from the L1 gene. The predicted protein sequences are derived by reference to U31781 and differ at 62 amino acid residues (2.4%), without nonsense substitutions. de Villiers et al. (12) recommend that a nucleotide divergence of 15% be used as the threshold for designation of a new type of human papillomavirus. The new strain is therefore well within the range of diversity expected within type 23 and has been designated HPV-23/Lancaster/2015. It is only the second full-length genome of HPV-23 to be described.

Forslund et al. (13) found HPV types of the *Betapapillomavirus 2* species in 9% of nasal swabs in a study of 312 Danish health care staff, but they detected HPV-23 in only 2 individuals (<0.7%). Of 9 deep-sequenced nonoverlapping subsets of individuals from our 51 swabs, we detected HPV-23 in all but one. Our frequency is therefore at least 8/51 (>15%) and possibly much higher. The significance of this clinically important papillomavirus at the prevalence in our sample remains a matter for speculation (BAM files, alignments, and phylogenetic trees are available from https://doi.org/10.17635/lancaster/researchdata/134).

### Accession number(s).

The genome sequence of HPV-23/Lancaster/2015 has been deposited in GenBank under the accession number KY652675.
